# The Effect of Statins on C-Reactive Protein in Stroke Patients: A Systematic Review of Clinical Trials

**DOI:** 10.1155/2021/7104934

**Published:** 2021-08-27

**Authors:** Babak Alikiaii, Zahra Heidari, Mohammad Bagherniya, Gholamreza Askari, Thozhukat Sathyapalan, Amirhossein Sahebkar

**Affiliations:** ^1^Anesthesia and Critical Care Research Center, Isfahan University of Medical Sciences, Isfahan, Iran; ^2^Department of Biostatistics and Epidemiology, School of Health, Isfahan University of Medical Sciences, Isfahan, Iran; ^3^Isfahan Cardiac Rehabilitation Research Center, Cardiovascular Research Institute, Isfahan University of Medical Sciences, Isfahan, Iran; ^4^Food Security Research Center, Isfahan University of Medical Sciences, Isfahan, Iran; ^5^Department of Community Nutrition, School of Nutrition and Food Science, Isfahan University of Medical Sciences, Isfahan, Iran; ^6^Academic Diabetes, Endocrinology and Metabolism, Hull York Medical School, University of Hull, Hull, UK; ^7^Biotechnology Research Center, Pharmaceutical Technology Institute, Mashhad University of Medical Sciences, Mashhad, Iran; ^8^Applied Biomedical Research Center, Mashhad University of Medical Sciences, Mashhad, Iran; ^9^School of Pharmacy, Mashhad University of Medical Sciences, Mashhad, Iran

## Abstract

**Background:**

Statins reportedly have anti-inflammatory effects aside from their lipid-lowering impact. We investigated the effects of statin therapy on the level of C-reactive protein (CRP) or highly sensitive CRP (hs-CRP), a liver-derived marker of systemic inflammation, among stroke patients.

**Methods:**

An online search was performed in Scopus, PubMed/MEDLINE, ISI Web of Science, and Google Scholar up to November 2020 to recognize clinical trials investigating the effects of statins on the CRP level in stroke patients.

**Results:**

Overall, nine studies (11 treatment arms) with 1659 participants met the inclusion criteria. Six out of 9 studies (8 out of 11 arms) were categorized as studies with a high-quality methodological approach using the Cochrane Collaboration's tool. Data from 5 treatment arms indicated a significant decrease in CRP concentration, and in one treatment arm, CRP concentration did not suggest any considerable alteration following statin therapy. Moreover, two treatment arms showed a significant reduction in hs-CRP concentration and three treatment arms revealed no significant alteration in hs-CRP concentration following statin therapy. Generally, results were heterogeneous and independent of the type of statin, statin dose, treatment duration, and changes in plasma low-density lipoprotein cholesterol concentration.

**Conclusion:**

The results suggest that statin therapy could reduce and, therefore, could be considered in these patients as potential anti-inflammatory agents.

## 1. Introduction

Stroke is a leading cause of severe and long-term disability and is considered the third common cause of human mortality. According to the estimates, annually, 15 million people suffer stroke worldwide, with an annual mortality rate of about 5 million [1]. High blood pressure and atrial fibrillation are the most important risk factors for stroke [2].

Ischemic stroke also referred to as brain ischemia or cerebral ischemia is the most common type of stroke, accounting for 80% of all cases [3]. The leading cause of ischemic stroke is the narrowing of the arteries due to atherosclerosis. However, there are many other causes of cerebral ischemic pathogenesis, including endothelial dysfunction, thrombogenesis, inflammatory and oxidative stress damages, and defects in angiogenesis [4].

Inflammatory damage plays a pivotal role in the pathogenesis of ischemic stroke. The collective contribution of the inflammatory cells in the ischemic tissue usually results in longstanding vascular inflammation and ischemic brain injury [5, 6]. Among various proinflammatory cytokines and mediators, serum C-reactive protein (CRP) is of particular importance. According to several studies, this protein is a neuroinflammation marker and an indicator of treatment efficacy [7]. Furthermore, studies have revealed that CRP could also be potentially used to predict impending atherosclerotic-related diseases, including ischemic stroke and cardiovascular disorders [8].

Statins are inhibitors of hydroxymethylglutaryl-coenzyme A (HMG-CoA) reductase, which have been proved to improve endothelial function, modulate thrombogenesis, and significantly diminish cardiovascular disorders [9–11]. In addition, the preventive and ameliorative effects of statins on myocardial infarctions and stroke have been thought to lower serum cholesterol levels [12]. However, it has been demonstrated that the inhibitory effects of statins on HMG-CoA reductase could result in pleiotropic effects beyond reducing the serum low-density lipoprotein (LDL) and cholesterol [13–20]. In this regard, it has been proved that statins could prevent ischemic stroke through attenuating inflammatory damage [21]. Moreover, various preclinical and clinical studies have reported the beneficial effect of statins on CRP reduction [22–24].

Despite the published clinical trials reporting the effects of statins on the CRP level in stroke patients, the findings of these studies have not been systematically reviewed. Therefore, we aimed to perform a systematic review of published clinical studies assessing the effects of statins on CRP levels in patients with stroke.

## 2. Material and Methods

This systematic review was designed and reported using the guidelines of the preferred reporting items for systematic reviews and meta-analyses (PRISMA) [25].

### 2.1. Search Strategy

We performed a conclusive systematic search on medical databases including Scopus, ISI Web of Science, PubMed, and Google Scholar databases from inception up to 12 November 2020 using the following keywords: (“statin therapy” OR “statin” OR “atorvastatin” OR “fluvastatin” OR “lovastatin” OR “pitavastatin” OR “pravastatin” OR “rosuvastatin” OR “simvastatin”) AND (“stroke” OR “Brain attack” or “Cerebrovascular accident” OR “CVA” OR “Hemorrhagic stroke” OR “Ischemic stroke”) AND (“CRP” OR “hs-CRP” OR “high sensitivity C-reactive protein” OR “C-reactive protein” OR “C-reactive protein”) AND (“Intervention Study” OR “Intervention Studies” OR “Controlled trial” OR “Randomized controlled trial” OR “Randomized clinical trial” OR “Non-Randomized Controlled Trials” OR “Clinical Trial” OR “Non-Randomized Controlled Trials” OR “Cross-Over study” OR “Cross-Over trial” OR “Cross Over trial” OR “Cross Over study” OR “Double-Blind Method” OR “Double-Blind” OR “Double-Blind trial” OR “Double-Blind study”). In addition, whenever possible, Medical Subject Headings (MESH) terms were used.

### 2.2. Study Selection

The title and abstract of all papers, which were found in early search, were independently reviewed by two authors (M.B. and G.A.). Articles that did not meet the inclusion criteria were excluded using a screen form with a hierarchical approach based on the study design, population, exposure, and outcome. To explore additional studies, reference lists of relevant review articles were reviewed. The full text of the eligible citation was reviewed. Any disagreements were discussed and agreed.

### 2.3. Inclusion Criteria

The search was conducted to identify articles examining the effects of statin therapy on CRP or hs-CRP in stroke patients. In the present systematic review, only original articles following these criteria were included: (1) using clinical trial design, (2) using statins as a drug, (3) conducted on patients with stroke disease as a primary disease, (4) assessing CRP or hs-CRP, and (5) using the English language.

### 2.4. Exclusion Criteria

Studies were excluded if they (1) were a nonhuman experimental disease, (2) reported duplicate data, and (3) were reviews, letters, editorial articles, study protocol, or case reports.

### 2.5. Data Extraction

Relevant articles were selected after screening records in the initial search. The following information was extracted from eligible and included articles and reported in Table 1: publication information including first author's last name, publication date, and study location, details of the clinical trial including target population, sample size, gender, the mean of age (years), study design, intervention (treatment), dose, control, duration of treatment, and main results of the studies.

### 2.6. Quality Assessment

The quality of the included studies was assessed by two independent researchers (M.B.) and (G.A.) using the Cochrane Collaboration's tool [26]. The following vital parts are included in this tool: random sequence generation, allocation concealment, blinding, incomplete outcome data, and selective reporting. Each item was categorized as low/unclear/high risk of bias. Consequently, if a study had more than two items of low risk, it was classified as a study with good quality. If a study had two items of low risk, it was considered a study with acceptable quality, and if a study had less than two items of low risk of bias, it was considered a study with weak quality [26].

## 3. Results

### 3.1. Search Results and Study Selection

**Figure 1:**
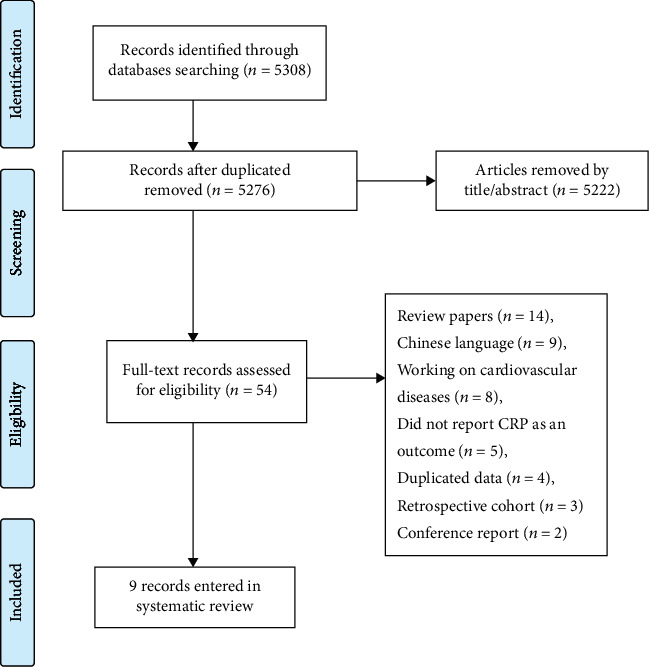
Flow chart of the process of the study selection.

A total of 5308 studies were acknowledged during the initial search, 5119 references in Scopus, 115 in PubMed, and 74 studies in the Web of Sciences, of which 32 records were duplicated. After reading the title and abstracts, 5222 irrelevant records were omitted. Full texts of these 54 remained articles were reviewed, and according to our inclusion and exclusion criteria, 45 articles were omitted due to the following reasons: review papers (*n* = 14), Chinese language (*n* = 9), working on cardiovascular diseases (*n* = 8), did not report CRP as an outcome (*n* = 5), duplicated data (*n* = 4), retrospective cohort (*n* = 3), and conference report (*n* = 2). The results of the search are shown in Figure 1. Thus, data extraction was done on nine articles with 11 arms. The characteristics of each selected paper are shown in Table 1.

### 3.2. Characteristics of the Included Studies

Included studies were published between 2004 and 2018 and were conducted in Japan (*n* = 1) [27], Italy (*n* = 2) [28, 29], China (*n* = 2) [30, 31], and Spain (*n* = 1) [32], and one each from Australia [33], India [34], and South Korea [35]. In total, 1659 participants with a mean age of 46–75 years were allocated to these studies and sample sizes ranged from 32 [35] to 1095 [27]. Eight studies included both men and women, and one study did not report the gender status of participants [33]. The duration of statin intervention ranged between 72 h [28] and six months [30]. Five studies used atorvastatin alone, three at the dose of 80 mg/day [28, 29, 33], one at the dose of 60 mg/day [31], and one at the dose of 10 mg/day [34]. One study used 10 mg/day of pravastatin alone [27], and one study used 20 mg/day of simvastatin alone [35]. Combined therapy was administered in 2 trials. One study used rosuvastatin 10 mg/day plus clopidogrel [30], and in one study, simvastatin, 20 and 40 mg/day, was used in combination with aspirin or triflusal [32]. Finally, five trials measured CRP [28–30, 34, 35] and four trials measured hs-CRP [27, 31–33].

### 3.3. Main Results

Changes in plasma CRP/hs-CRP concentrations following statin therapy were reported in 11 treatment arms. Data of 5 treatment arms indicated a significant decrease in CRP concentration [28, 30, 34, 35], and one treatment arm CRP concentration did not reveal any significant alteration following statin therapy [29]. Moreover, two treatment arms showed a significant reduction in hs-CRP concentration [27, 31] and the three treatment arms revealed no significant alteration in hs-CRP concentration following statin therapy [32, 33].

When the included studies were arranged according to the type of statin used, CRP concentration in 3 treatment arms was decreased [28, 34], and in one arm, it did not alter after atorvastatin therapy [29]. On the other hand, hs-CRP concentration in one study arm decreased after atorvastatin monotherapy [31]. In 2 treatment arms, alteration in hs-CRP concentration was not significant when single doses of atorvastatin were used [33]. In a different arm, hs-CRP concentration significantly decreased after monotherapy of pravastatin [27]. A significant reduction in CRP concentration was observed after monotherapy of simvastatin in one treatment arm [35], but combination therapy of simvastatin with aspirin or triflusal did not alter the hs-CRP concentration in another study arm [32]. Finally, CRP concentration significantly decreased in one study arm after using rosuvastatin combined with clopidogrel [30].

Overall, statin therapy with lower doses was more effective than higher doses. A significant reduction in CRP/hs-CRP concentrations was observed upon administration of low-dose statin treatments. There was no association between the duration of statin therapy and changes in plasma CRP/hs-CRP concentrations.

### 3.4. Quality of the Included Studies

As shown in Table 2, 6 out of 9 studies had a high-quality methodological approach [28–33], one study was categorized as a study with acceptable quality [27], and two studies had a weak methodological design [34, 35].

## 4. Discussion

This review is aimed at systematically and comprehensively evaluating the evidence regarding the effect of statin on CRP in patients with stroke to provide the groundwork for future studies. To our knowledge, this systematic review is the first to assess the association between statin therapy and plasma CRP concentrations among patients with stroke. As described in the results, since the population of statin-treated patients and follow-up durations were heterogeneous across included studies, we did not conduct a meta-analysis. However, the main finding of the current qualitative study is that statin therapy in stroke patients is associated with reducing CRP as an acute-phase reactant and sensitive marker of systemic inflammation. Furthermore, the role of inflammation as a triggering factor for increasing blood viscosity, promoting plaque formation, and accelerating atherosclerosis, through CRP, tumor necrosis factor-alpha (TNF-*α*), and interleukin 6 (IL-6), as nonspecific markers of the acute stage of the systemic inflammatory response, is well established. Thus, there is a complementary relationship between the level of inflammation and atherosclerotic plaque formation [30, 34, 36].

Although the beneficial effects of statins are mediated predominantly by their lipid-lowering effects, recent evidence from clinical trials suggests that statins have anti-inflammatory effects and their benefits may extend beyond their cholesterol-lowering effects that are important for prognosis and treatment in cardiovascular and stroke events [34, 37, 38]. Statin therapy has suggested protecting vascular events through anti-inflammatory activities reflected by CRP reductions [32, 34]. Growing evidence of CRP-reducing effects of statins indicates that this class of medications reduces the risk of cardiovascular events in patients with coronary artery disease [37, 39–41]. In this regard, Ridker et al. demonstrated that statin therapy resulted in a more significant clinical benefit when CRP levels were high and that statins decreased CRP levels in a manner essentially independent of LDL-C levels [42].

It is well documented that the induction of an inflammatory response plays an essential role in the pathogenesis of brain damage. For example, elevated serum levels of CRP in the inflammatory process of atherosclerosis have been widely considered to increase intimal thickness and plaque rupture, resulting in acute cerebral infarction [30, 31]. This phenomenon implies that the onset and development of atherosclerotic lesions could be modulated by reducing inflammation. In this regard, anti-inflammatory therapies are neuroprotective and preventing neuroinflammation may add a better clinical outcome to ischemic stroke [30, 43].

In addition to their effects in reducing cardiovascular risk, there is increasing evidence that prior or early use of statins may reduce the severity of an acute ischemic stroke and improve its outcome [22, 32, 43–46]. It has been shown that statins stabilize atherosclerotic plaque and increase cerebral blood flow [30, 44]. Data from several systematic reviews and meta-analyses support statins' benefit in stroke patients [47–52]. In several trials, statin therapy has been shown to significantly reduce the plasma level of CRP and patients with any stroke history who have low CRP levels after statin therapy have better clinical outcomes than those with higher CRP levels [27, 34, 43, 53, 54].

In the present review, only two studies were designed to directly assess the effects of statins on CRP levels in stroke patients due to their pleiotropic activities [27, 34]. However, the results of these two studies showed a significant reduction in CRP concentrations after statin therapy, confirming the potential anti-inflammatory effects of statins aside from their cholesterol-lowering impact. Moreover, in noncardiogenic ischemic stroke patients, pravastatin decreased the hs-CRP levels and elevated hs-CRP levels were suggested to increase the risk of recurrent stroke and vascular events [27].

In two studies, the effect of statins on the inflammatory markers in stroke patients was evaluated. In a randomized parallel trial, atorvastatin 80 mg/day acutely administered immediately after an atherosclerotic ischemic stroke was found to reduce serum levels of inflammatory markers including CRP, confirming the neuroinflammatory protection of statins [28]. In another trial, atorvastatin and rosuvastatin revealed lipid-lowering and anti-inflammatory effects in stroke patients and reduced CRP levels, although rosuvastatin showed better therapeutic benefits [30]. In addition, in other trials, CRP was also measured after statin usage in stroke patients. These studies showed a decrease in plasma CRP concentration in stroke patients after using statins [31, 35].

In contrast to the above studies, there have also been studies in which statin therapy did not affect the concentrations of CRP in stroke patients. In the study of Montaner and coworkers, assessment of inflammatory markers, including CRP, after using simvastatin did not show any difference in their levels regarding treatment allocation and authors report a nonsignificant increase in mortality and a more significant proportion of infections in the simvastatin group as the primary safety concerns [32]. Moreover, two independent studies demonstrated that administration of atorvastatin 80 mg/day did not affect the concentration of CRP in stroke patients. Muscari and coworkers observed a lack of increase in the CRP level in the atorvastatin group, compared with the significant increase in the placebo group. They conclude that this difference was likely due to the anti-inflammatory effect of atorvastatin [29]. In the second study, atorvastatin prescription for 3 and 30 days did not reduce CRP in stroke patients and did not appear to modify infarct growth substantially [33].

Although some studies did not meet our inclusion criteria, their results concerning statins' effect on CRP levels were interesting. Notably, in two studies, reducing CRP levels following statin therapy was significantly associated with favorable 3-month outcomes and improved patients' survival and readmission rates after acute ischemic stroke [43, 53]. Moreover, in one retrospective study, patients with a history of stroke after prescribing pitavastatin showed a reduced CRP level and potentially limited atherosclerosis in high-risk stroke patients [54]. Furthermore, in the Justification for the Use of Statins in Primary Prevention (JUPITER) trial, a study conducted on healthy subjects without hyperlipidemia and with elevated CRP levels; rosuvastatin 20 mg was administered daily and was shown to decrease both LDL cholesterol and CRP levels, reducing the occurrence of ischemic stroke [55]. In our systematic review, the association between statin therapy and CRP levels did not vary upon the statin type and dose.

**Figure 2:**
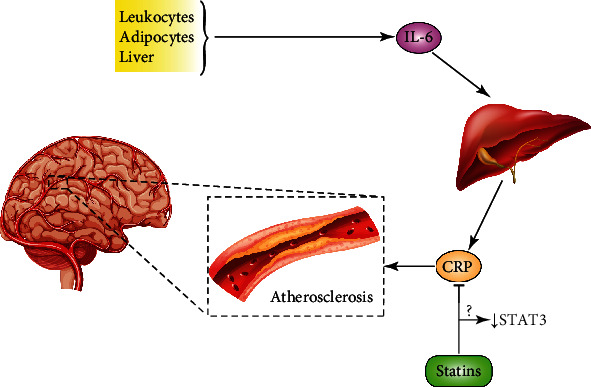
Potential mechanisms leading to statin-induced reduction of CRP release from hepatocytes in stroke. Statins reduce activation of transcription factor STAT3, leading to a decrease of CRP release by hepatocytes. Reduction of the CRP level attenuates inflammatory response and eventually leads to the neuroprotective effects during the stroke.

The mechanisms by which statins reduce CRP levels in stroke patients are not precisely known. CRP is synthesized mainly by the liver in response to proinflammatory cytokines, particularly IL-6 derived from activated leukocytes, adipose tissue, and in part from the liver [56]. After releasing in the bloodstream, CRP induces upregulation of the vasoconstrictor endothelin-1 and IL-6 by endothelial cells and increases vascular cell adhesion molecule-1, intercellular adhesion molecule-1, E-selectin, and monocyte chemoattractant protein-1 (MCP-1) and thus enhances leukocyte recruitment in an inflammatory process [38]. As depicted in Figure 2, statins possibly reduce IL-6-induced CRP expression in human hepatocytes. At the transcriptional level, statins act on the geranylgeranyl pathway and decrease activation of the transcription factor STAT3, leading to the attenuation of inflammation and neuroprotection effects after stroke.

Several potential limitations to this study should be noted. First, the total number of studies was limited and the trial population size was relatively small. Second, because studies with almost high heterogeneity were included in this study, estimates from these studies could not be reliably combined to conduct a meta-analysis. Finally, this heterogeneity made us interpret our results cautiously. Differences in the duration of treatment, statin dose, control group treatment, and design of studies were some reasons for potential heterogeneity across studies.

Furthermore, various changes in plasma CRP concentrations are dependent on different pharmacokinetic profiles of statins used. Third, we included only studies published in English and might have missed relevant articles in languages other than English. Finally, if the literature search fails to find all relevant reports, the present study is at risk of bias.

## 5. Conclusion

Our research showed the beneficial impact of statins in patients after a stroke by reducing CRP levels confirming available evidence regarding the potential benefits of statins as anti-inflammatory agents. These findings could propose that statin treatment should be started in patients with stroke, irrespective of their levels of cholesterol; however, well-designed trials in patients with stroke are needed to precisely examine the CRP-reducing benefits of statin therapy in the future, considering potential differences by dosage, duration of use, study population, and other factors.

## Figures and Tables

**Table 1:** Summarize the studies included in the systematic review (arranged alphabetically by first author's last name).

ID	First author, country, year	Target population	Sample size (intervention, control)	Gender (male/female)	Age (mean ± SD)	Study design	Intervention	Dose	Control	Duration	Main results
1	Antonino Tuttolomondo, Italy, 2016 [28]	Acute ischemic stroke	42 (22/20)	23/19	66.27 ± 19.34	Randomized parallel trial	Atorvastatin	80 mg/day	No treatment	72 h	CRP ↓
2	Antonino Tuttolomondo, Italy, 2016 [28]	Acute ischemic stroke	42 (22/20)	23/19	66.27 ± 19.34	Randomized parallel trial	Atorvastatin	80 mg/day	No treatment	7 days	CRP ↓
3	Christopher Beer, Australia, 2012 [33]	Acute ischemic stroke	40 (20/20)	NM	68.6 ± 13.8	Randomized parallel trial	Atorvastatin	80 mg/day	Placebo	3 days	hs-CRP ↔
4	Christopher Beer, Australia, 2012 [33]	Acute ischemic stroke	38 (17/21)	NM	68.6 ± 13.8	Randomized parallel trial	Atorvastatin	80 mg/day	Placebo	30 days	hs-CRP ↔
5	Antonio Muscari, Italy, 2011 [29]	Ischemic stroke	62 (31/31)	20/42	75.3 ± 11.9	Double-blind, placebo-controlled, parallel group study	Atorvastatin	80 mg/day	Placebo	7 days	CRP ↔
6	Xingyu Chen, China, 2018 [31]	Acute ischemic stroke	117 (60/57)	73/43	61.67 ± 11.67	Preliminary, randomized controlled	Atorvastatin	60 mg/day	Atorvastatin 20 mg/day	7 days	hs-CRP ↓
7	Kazuo Kitagawa, Japan, 2017 [27]	Non-cardiogenic ischemic stroke	1095 (545/550)	755/340	66.2 ± 8.5	Randomized open-label trial	Pravastatin	10 mg/day	No treatment	2 months	hs-CRP ↓
8	Jae-Kwan Cha, South Korea, 2004 [35]	Atherosclerotic ischemic stroke	32 (32/0)	28/4	68.5	Trial	Simvastatin	20 mg/day	No control group	12 weeks	CRP ↓
9	Joan Montaner, Spain, 2008 [32]	Cortical stroke	56 (28/28)	29/27	72.7 ± 12.6	Pilot, double-blind, randomized, multicenter clinical trial	Simvastatin + aspirin or simvastatin + triflusal	Simvastatin 40 mg/day firs week 20 mg/day until day 90 aspirin 300 mg/day or triflusal 900 mg/day and followed with aspirin 300 mg/day or triflusal 600 mg/day until day 90	Placebo + aspirin or placebo + triflusal	90 days	hs-CRP ↔
10	A. Vijaya Anand, India, 2009 [34]	Stroke	95 (35/60)	64/31	60.1 ± 7.4	Clinical controlled trial	Atorvastatin	10 mg/day	No treatment	3 months	CRP ↓
11	Guo-jun Cao, China2017 [30]	Cerebral infarction	120 (60/60)	65/55	46.29 ± 7.48	Randomized parallel trial	Clopidogrel (75 mg) + rosuvastatin 10 mg/day	10 mg/day	Clopidogrel (75 mg) + atorvastatin 20 mg/day	6 months	CRP ↓

**Table 2:** Risk of bias assessment for included clinical trials.

First author (publication year)	Random sequence generation	Allocation concealment	Blinding of participants and personnel	Blinding of outcome assessment	Incomplete outcome data	Selective reporting	Other risks of bias
Antonino Tuttolomondo (2016)	L	L	H	H	L	L	U
Christopher Beer (2012)	L	L	L	L	U	L	H
Antonio Muscari,(2011)	L	L	L	L	L	L	U
Xingyu Chen (2018)	L	L	L	L	L	L	L
Kazuo Kitagawa (2017)	L	L	H	H	U	U	H
Jae-Kwan Cha (2004)	H	H	H	H	H	L	U
J. Montaner (2007)	L	L	L	L	L	L	U
Vijaya Anand (2009)	H	H	U	U	U	U	U
Guo-jun Cao (2017)	L	L	U	U	L	L	U

L: low risk of bias; H: high risk of bias; U: unclear risk of bias.

## Data Availability

There is no raw data associated with this systematic review.
